# A dual-modality workflow for quantifying microvascular structure in human temporal lobe epilepsy

**DOI:** 10.1016/j.ibneur.2026.08.017

**Published:** 2026-08-21

**Authors:** Ziyu Xu, Vasiliki Tempeli, Steffanie Hartjes, Alexander Kotenko, Govert Hoogland, Roel H.L. Haeren, Olaf E.M.G. Schijns, Rick H.G.J. van Lanen, Marc A.M.J. van Zandvoort, Kim Rijkers, Dimitrios Kapsokalyvas, on behalf of the ACE Epilepsy Surgery group

**Affiliations:** a Department of Genetics & Cell Biology, Maastricht University, Maastricht, the Netherlands; b Mental Health and Neuroscience Research Institute (MHeNS), Maastricht University, Maastricht, the Netherlands; c Department of Neurosurgery, Maastricht University Medical Center, Maastricht, the Netherlands; d Academic Centre for Epileptology (ACE), Maastricht University Medical Centre and Kempenhaeghe, Heeze, Maastricht, the Netherlands; e Cardiovascular Research Institute Maastricht (CARIM), Research Institute for Oncology and Reproduction (GROW), Maastricht University, Maastricht, the Netherlands; f Institute for Molecular Cardiovascular Research (IMCAR), University Hospital RWTH Aachen, Aachen, Germany; g Center for Integrative Neuroscience Maastricht (CIN), Maastricht University, Maastricht, the Netherlands; h Light Microscopy Facility, Interdisciplinary Center for Clinical Research (IZKF), University Hospital RWTH Aachen, Aachen, Germany

**Keywords:** Temporal lobe epilepsy, Microvascular remodeling, Lipofuscin, Spectral unmixing, Hippocampal sclerosis, Automated morphometry

## Abstract

Microvascular remodeling is implicated in the pathophysiology of drug-resistant temporal lobe epilepsy (TLE). However, characterizing these changes in adult human surgical tissue is methodologically limited by the accumulation of lipofuscin, an autofluorescent pigment that obscures microvascular structures and confounds standard automated quantification. To address this, we developed a dual-modality workflow optimized for archival human tissue obtained from drug-resistant TLE patients and one post-mortem control. This approach integrates hematoxylin and eosin (H&E) staining for precise anatomical subfield delineation with lectin-based fluorescence imaging and spectral unmixing, a computational technique that separates specific vascular signals from overlapping lipofuscin autofluorescence. Using QuPath, an open-source image analysis platform, we validated the accuracy of this automated workflow by comparing the microvascular area fraction (MAF) against manual ground-truth annotations in randomized regions of interest. The automated workflow demonstrated excellent concordance with manual assessment (Spearman's ρ = 0.997, p < 0.001). Application of this method to a cohort of TLE patients revealed substantial inter-patient heterogeneity. Exploratory analysis showed a positive but non-significant association between hippocampal MAF and preoperative seizure frequency (ρ = 0.68, p = 0.14), suggesting a possible relationship that requires confirmation in larger cohorts. Furthermore, the workflow successfully resolved subfield-specific vascular heterogeneity within sclerotic tissue that is typically obscured by whole-region average morphometry. This study establishes a robust, spectrally-unmixed morphometry pipeline that effectively resolves lipofuscin artifacts in adult human brain tissue, providing a necessary methodological foundation for future pathology-stratified investigations into cerebrovascular remodeling in epilepsy.

## 1 Introduction

Epilepsy is one of the most prevalent neurological disorders globally, affecting over 70 million people worldwide (Thijs et al., 2019). Temporal lobe epilepsy (TLE) is the most frequent subtype of focal epilepsy (Téllez-Zenteno and Hernández-Ronquillo, 2012), and approximately 30% of TLE patients develop drug-resistant epilepsy (DRE) (Zuberi et al., 2022). In adults, the most common pathological substrate for drug-resistant TLE is hippocampal sclerosis (HS), characterized by selective neuronal loss and gliosis (Blumcke et al., 2017; Thom, 2014). For a carefully selected subgroup of patients, resective surgery is an evidence-based, potentially curative treatment option, resulting in seizure freedom in 70–85% of cases (Wiebe and Jetté, 2012). For patients who are not eligible for surgical intervention, improved seizure control depends on the development of novel treatment approaches, which requires a better understanding of the underlying pathophysiology.

In this regard, there is increasing interest in the role of the cerebral microcirculation in epilepsy pathophysiology. Under physiological circumstances, the cerebral microcirculation regulates blood flow, nutrient delivery, and metabolite clearance (Iadecola, 2017). Emerging evidence suggests that microvascular abnormalities contribute to epileptogenesis through multiple mechanisms, including blood-brain barrier (BBB) dysfunction, increased vascular permeability, and microvascular remodeling (Muoio et al., 2014; Rigau et al., 2007; van Lanen et al., 2021). BBB disruption permits albumin extravasation, which induces astrocytic responses and neuronal hyperexcitability (Ivens et al., 2007; Reiss et al., 2023). Microvascular remodeling, including altered vessel density and compromised cerebrovascular autoregulation, has been observed in both epilepsy patients and experimental models (Lim et al., 2021; Rigau et al., 2007). Whether these changes are a cause or consequence of seizures remains unclear, but neurovascular alterations are now recognized as playing an important role in the pathophysiological cascade underlying epilepsy (van Lanen et al., 2021).

Characterizing microvascular changes in human surgical tissue is methodologically challenging and has yielded conflicting findings. Some studies report reduced vascular density and increased string vessels (Kastanauskaite et al., 2009, Mott et al., 2009), whereas others describe angiogenesis and increased density. These discrepancies likely reflect methodological heterogeneity in vascular labeling, sampling strategies, and analysis pipelines (van Lanen et al., 2021). Recent advances in automated whole-slide imaging using open-source platforms such as QuPath have successfully quantified microvessels in mouse models (Bankhead et al., 2017), offering a robust alternative to manual counting. However, translating these workflows to adult human tissue is complicated by lipofuscin, an autofluorescent pigment that accumulates in lysosomes due to age and metabolic stress (Dougnon and Matsui, 2025a; Sun et al., 2017). Its broad emission spectrum overlaps with common vascular markers, generating false-positive signals that confound automated segmentation (Dougnon and Matsui, 2025b). Although chemical quenching methods such as Sudan Black B can reduce lipofuscin autofluorescence, they may also attenuate specific immunofluorescent signals and compromise tissue integrity (Sapio et al., 2025).

To address these limitations, we developed a dual-modality workflow optimized for archival human TLE tissue. This approach combines hematoxylin and eosin (H&E) staining for anatomical subfield delineation with lectin-based fluorescence imaging and spectral unmixing, a computational technique that separates specific vascular signals from overlapping lipofuscin autofluorescence (Sadashivaiah et al., 2023). The present study validates this workflow as a robust tool for quantifying the microvascular area fraction (MAF) in drug-resistant TLE and demonstrates its capacity to resolve subfield-specific vascular heterogeneity often obscured by traditional quantification methods.

## 2 Materials and methods

### 2.1 Ethical considerations

This study was approved by the local medical ethics committee (METC azM/UM, ID METC 2018–0664) and complies with the Declaration of Helsinki and Good Clinical Practice.

### 2.2 Tissue collection and preparation

We included drug-resistant TLE patients who underwent amygdalohippocampectomy (AH) with or without anterior temporal lobectomy (ATL) as a treatment for their epilepsy. Hippocampal sclerosis (HS) was histopathologically confirmed according to the International League Against Epilepsy (ILAE) classification system (Blümcke et al., 2013). Neocortical and hippocampal tissue samples were collected intraoperatively, fixed in 4% paraformaldehyde (PFA) for 48 h, and embedded in paraffin.

Post-mortem control neocortical and hippocampal tissue was obtained with a post-mortem interval (PMI) of 6 h. After 48 h fixation in 4% PFA and vacuum infiltration processing (VIP) dehydration, the specimen was embedded in paraffin and stored at room temperature.

Paraffin-embedded tissue blocks were sectioned at 5 μm using a rotary microtome (Leica, Model 2045), mounted on Polysine glass slides (Gerhard Menzel GmbH), and dried overnight at 37 °C.

The clinical and demographic characteristics of the study cohort are presented in Table 1 (see 3.1).

**Table 1:** Clinical and demographic characteristics of the study cohort.

**Patient**	**Tissue**	**Age (y)**	**Sex**	**BMI**	**Onset (y)**	**Dur. (y)**	**Sz. Freq.**	**History**	**HS**	**Hip MAF (%)**
**P1**	None*	46	F	27	20	26	Weekly	None	+	N/A
**P2**	None*	50	F	26	1	49	Weekly	Thyroid	+	N/A
**P3**	HIP	53	F	26	43	10	Monthly	None	−	0.55
**P4**	None*	55	F	23	40	15	Weekly	None	−	N/A
**P5**	HIP	40	M	23	20	20	Weekly	TBI, Migraine	−	0.50
**P6**	HIP	33	F	32	31	2	> 1/Day	None	+	2.35
**P7**	HIP & NC	28	F	22	25	3	> 1/Day	CI	+	0.80
**P8**	HIP & NC	37	F	23	20	17	Daily	None	−	1.61
**P9**	HIP & NC	41	M	22	12	29	Daily	TBI	−	1.11
**P10**	None*	52	F	33	37	15	Weekly	DVT/PE	+	N/A
**Control**	HIP & NC	52	N/A	N/A	N/A	N/A	N/A	N/A	N/A	0.65

**Abbreviations:** Dur., duration of epilepsy; Sz. Freq., seizure frequency; HS, hippocampal sclerosis (histopathologically confirmed: +, positive; −, negative; N/A, not applicable); Hip, hippocampus; MAF, microvascular area fraction. History includes relevant comorbidities: TBI, traumatic brain injury; CI, cerebral infection; DVT/PE, deep vein thrombosis/pulmonary embolism (Factor V Leiden deficiency). Weekly, 2–6 seizures/week; Monthly, 1 seizure/month; Daily, 1 seizure/day; > 1/Day, more than one seizure per day. "−" in other columns indicates data not available. Patients P1, P2, P4, and P10 (Tissue = None) were excluded from automated microvascular quantification due to suboptimal histological quality.

### 2.3 Staining

For histochemical staining, slides were deparaffinized in xylene and rehydrated through a graded ethanol series, followed by rinsing in Milli-Q water. Hematoxylin and eosin (H&E) staining was performed on 5 μm sections following a standard protocol (Fischer et al., 2008; Titford, 2005), including brief rinses, graded ethanol dehydration, and xylene clearing.

For fluorescence staining, sections were permeabilized three times in Tris-buffered saline (TBS) with 0.05% Triton X-100 (TBS-T) for 5 min in glass Coplin jars. Sections were then incubated with Ulex europaeus agglutinin I conjugated to DyLight™ 594 (UEA I-DyLight™ 594; Vector Laboratories, Cat. No. DL-1067) at 10 μg/ML in TBS for 1 h at room temperature in the dark. After washing five times for 5 min each in TBS-T, sections were incubated with 4′,6-diamidino-2-phenylindole (DAPI; 100 ng/ML in TBS; Roche Diagnostics) for 20 min at room temperature in the dark. Following three washes of 5 min each in TBS, samples were mounted with ProLong Diamond Antifade (Cat. # P36961; ThermoFisher), dried at 37 °C for 5 min, and stored at 4 °C in the dark until imaging.

### 2.4 Imaging

Entire tissue sections were imaged to obtain a comprehensive overview of each sample. Adjacent serial sections were used: one stained with H&E and imaged under brightfield microscopy, and the other fluorescently labeled and imaged with fluorescence microscopy. Both modalities were acquired on an Olympus BX51WI microscope equipped with an electron-multiplying charge-coupled device (EM-CCD) camera (C9100; Hamamatsu Photonics Europe GmbH) for fluorescence and an RGB camera for brightfield. UEA I-DyLight 594 was imaged in the red channel (TRITC filter set), lipofuscin autofluorescence in the green channel (FITC filter set), and nuclei (DAPI) in the blue channel (DAPI filter set). Exposure times were set to 300 ms for the red and green channels and 30 ms for the blue channel. Sensitivity and offset were set to zero. Neurolucida software (version 2020; MBF Bioscience) was used for image acquisition. Samples were focused using a 2x objective (PlanApoN; 2x/0.08), and images were acquired using a 10x objective (UPlanSApo; 10x/0.40) with the stitching workflow enabled. Manual focus adjustments were made every four images, with blending set at 20 pixels per seam and trimming at 10 pixels.

### 2.5 Image processing

To address spectral crosstalk between lipofuscin autofluorescence and UEA I-DyLight 594 signals, we applied spectral unmixing using the Poisson non-negative matrix factorization (NMF) plugin (McRae et al., 2019) in Fiji (ImageJ v2.3.0/1.53q) (Neher et al., 2009). Reference emission profiles were empirically determined from regions containing pure lipofuscin autofluorescence and pure UEA I-DyLight 594-stained vessels, respectively.

Images were denoised using rolling-ball background subtraction (radius = 50 pixels) via the Subtract Background function. Contrast optimization was achieved through contrast-limited adaptive histogram equalization (CLAHE; block size = 255, histogram bins = 256, maximum slope = 3.0).

### 2.6 Vessel quantification

Microvascular analysis was performed using QuPath (v0.3.2) (Bankhead et al., 2017) on the single-channel unmixed images. The pixel-classification and segmentation functions used in this study are stable across subsequent QuPath releases (v0.4–v0.5), and no version-dependent differences in output are expected. Regions of interest (ROIs) were manually delineated using the wand tool to exclude areas with tissue folding or suboptimal vessel staining. For each tissue sample, a customized threshold was generated using the pixel classification function at full resolution (0.81 μm/pixel), with a Gaussian prefilter (smoothing sigma = 0.5) to reduce background noise. A minimum size threshold of 10 pixels (approximately 8 μm) was applied to filter out isolated noise pixels and debris in the red channel.

Annotated objects corresponding to the microvasculature were segmented and quantified. Shape descriptors were extracted using the calculate features function, and parameters including area and length were obtained via the show annotation measurements tool. The resulting data (total vessel area, total sample area, and vessel count) were exported for subsequent statistical analysis. All image annotations and parameter settings were applied consistently across samples using a standardized workflow to ensure reproducibility and minimize user bias. ROI selection was performed by trained researchers following an internally standardized protocol.

For the control specimen, the spectral characteristics of lipofuscin differed from those observed in the surgical TLE samples, likely reflecting differences in tissue processing (post-mortem fixation vs. intraoperative collection) and the distinct metabolic history of non-epileptic tissue. As a result, the NMF-based spectral unmixing did not achieve adequate separation of vascular and lipofuscin signals in this specimen. Vascular segmentation of the control tissue was therefore performed using a machine learning-based approach (artificial neural network multilayer perceptron, ANN-MLP) implemented through QuPath's built-in classifier module. The ANN-MLP was trained on a curated dataset containing manually annotated vessel masks and non-vascular regions delineated independently by two researchers blinded to each other's annotations, achieving a classification accuracy of 85% or greater (assessed by pixel-level agreement between automated and manual masks) (Bai et al., 2023; Saha Tchinda et al., 2021).

Microvascular area fraction (MAF, %) was calculated using the following formula:

(1) $$\mathrm{Microvascular}\;\mathrm{area}\;\mathrm{fraction}\;\left(\%\right)=\frac{\mathrm{Total}\;\mathrm{vessel}\;\mathrm{area}}{\mathrm{Total}\;\mathrm{sample}\;\mathrm{area}}\times 100\%$$

Total vessel area is the area of the UEA I-DyLight 594 positive vascular mask and total sample area is the area of the tissue mask within the ROI after excluding tears, folds, and empty background.

Mean vessel segment length (MVL, μm) was determined by centerline extraction (two-dimensional skeletonization) of the vascular mask, followed by measurement of each connected segment length. MVL represents the mean of all segment lengths within each ROI.

Mean vessel diameter (MVD, μm) was estimated at each centerline point as twice the local inscribed radius derived from the distance transform. MVD represents the mean diameter across all centerline points within each ROI.

All metrics were computed per ROI and aggregated per region and patient as described in 2.8.

### 2.7 Methodological validation

To validate the accuracy of the automated segmentation workflow, a subset of 10 ROIs was randomly selected from the hippocampal fluorescence dataset. Microvessels within these regions were manually delineated by an experienced researcher blinded to the results of the automated analysis. Concordance between manual ground-truth masks and automated segmentation masks was assessed for two metrics: vessel count and MAF. Agreement was quantified using Spearman's rank correlation coefficient.

### 2.8 Statistical analysis

Statistical analyses were performed using GraphPad Prism (version 9.0). For quantitative microvascular metrics (Fig. 4), individual patient values are displayed as strip plots with median lines; the post-mortem control is shown separately. For subfield-resolved comparisons (Fig. 6E), data are presented as mean ± standard deviation (SD) across regions of interest (ROIs) within each subfield. Due to the limited sample size, only non-parametric analyses were conducted. Validation of the automated segmentation workflow against manual ground-truth annotations was assessed using Spearman's rank correlation coefficient (ρ) across 10 randomized ROIs. To explore potential clinical associations, hippocampal MAF was treated as a continuous variable. Exploratory Spearman rank correlation analyses were performed to investigate the relationship between hippocampal MAF and clinical metadata, including preoperative seizure frequency, age at surgery, and epilepsy duration. Preoperative seizure frequency was categorized based on the average frequency documented during presurgical evaluation using an ordinal scale: Monthly (approximately one seizure per month), Weekly (two to six seizures per week), Daily (approximately one seizure per day), and > 1/Day (more than one seizure per day). A p-value < 0.05 was considered statistically significant, though all correlations were interpreted with caution given the limited sample size (n = 6 for hippocampal analyses). No formal group comparisons were performed due to insufficient statistical power.

## 3 Results

### 3.1 Study cohort

The clinical and demographic characteristics of the study cohort and the post-mortem control specimen are summarized in Table 1. Ten patients with drug-resistant TLE were initially enrolled. Four patients (P1, P2, P4, and P10) were excluded from automated microvascular quantification due to suboptimal histological quality. The final analytical cohort comprised six patients with hippocampal tissue, three of whom also had neocortical tissue available for quantitative analysis. The six analyzed patients comprised four females and two males, with a median age at surgery of 38.5 years (range 28–53 years). Seizure frequency ranged from monthly to more than one seizure per day. Two of the six patients had histopathologically confirmed hippocampal sclerosis.

### 3.2 Validation of the dual-modality workflow

**Fig. 1:**
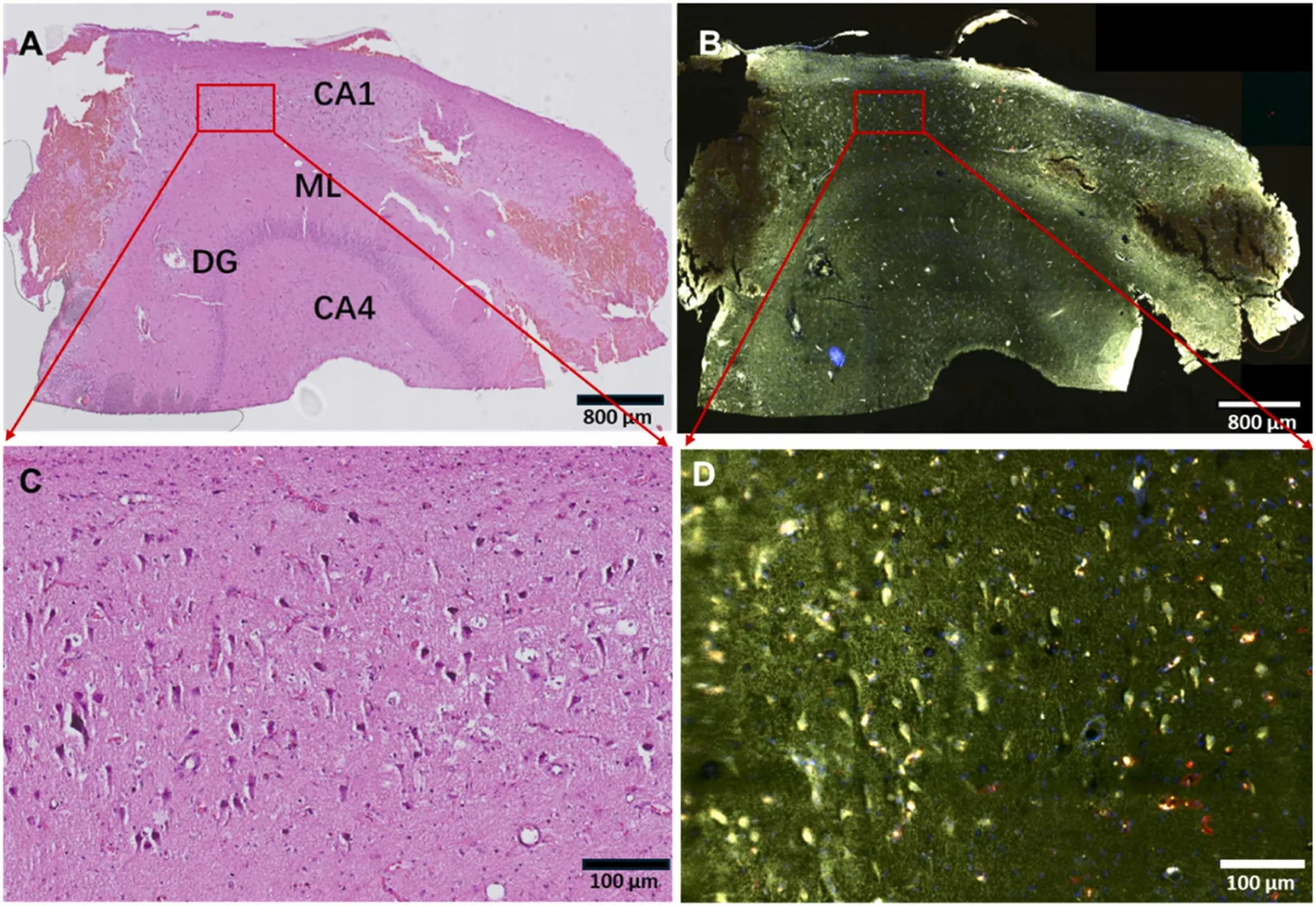
Dual-modality imaging of hippocampal tissue from a patient with hippocampal sclerosis (P6) (A) H&E-stained overview showing hippocampal subfield architecture. Anatomical regions are labeled: CA1 (Cornu Ammonis 1), ML (Molecular Layer), DG (Dentate Gyrus), and CA4 (Cornu Ammonis 4). Yellow box indicates the region shown at higher magnification in (C). Scale bar: 800 μm. (B) Composite fluorescence image of an adjacent section from the same specimen, stained with UEA I (DyLight 594, red), DAPI (blue), and showing lipofuscin autofluorescence (green). Yellow box indicates the corresponding region shown in (D). Scale bar: 800 μm. (C) High-magnification H&E image of the CA1 subfield. Note the reduced cellular density consistent with neuronal loss in hippocampal sclerosis. Scale bar: 100 μm. (D) Corresponding high-magnification fluorescence image of the same CA1 region. Abundant lipofuscin autofluorescence (green/yellow granular signal) is visible throughout the tissue, obscuring the specific vascular signal (red, UEA I) and illustrating the challenge of automated vessel segmentation in adult human brain tissue without spectral unmixing. Scale bar: 100 μm.

To address the challenges of quantifying microvasculature in archival tissue, we established a dual-modality workflow that co-registers standard H&E staining with lectin-based fluorescence imaging on adjacent sections (Fig. 1A, B). H&E-guided anatomical delineation enabled precise identification of hippocampal subfields, including CA1, CA4, and the dentate gyrus (Fig. 1A). High-magnification comparison of the same CA1 region under both modalities revealed the extent of lipofuscin autofluorescence interference in the fluorescence channel (Fig. 1C, D). The abundant green/yellow granular signal from lipofuscin deposits obscured the specific vascular marker (UEA I-DyLight 594), underscoring the necessity for computational spectral unmixing prior to automated vessel quantification.

**Fig. 2:**
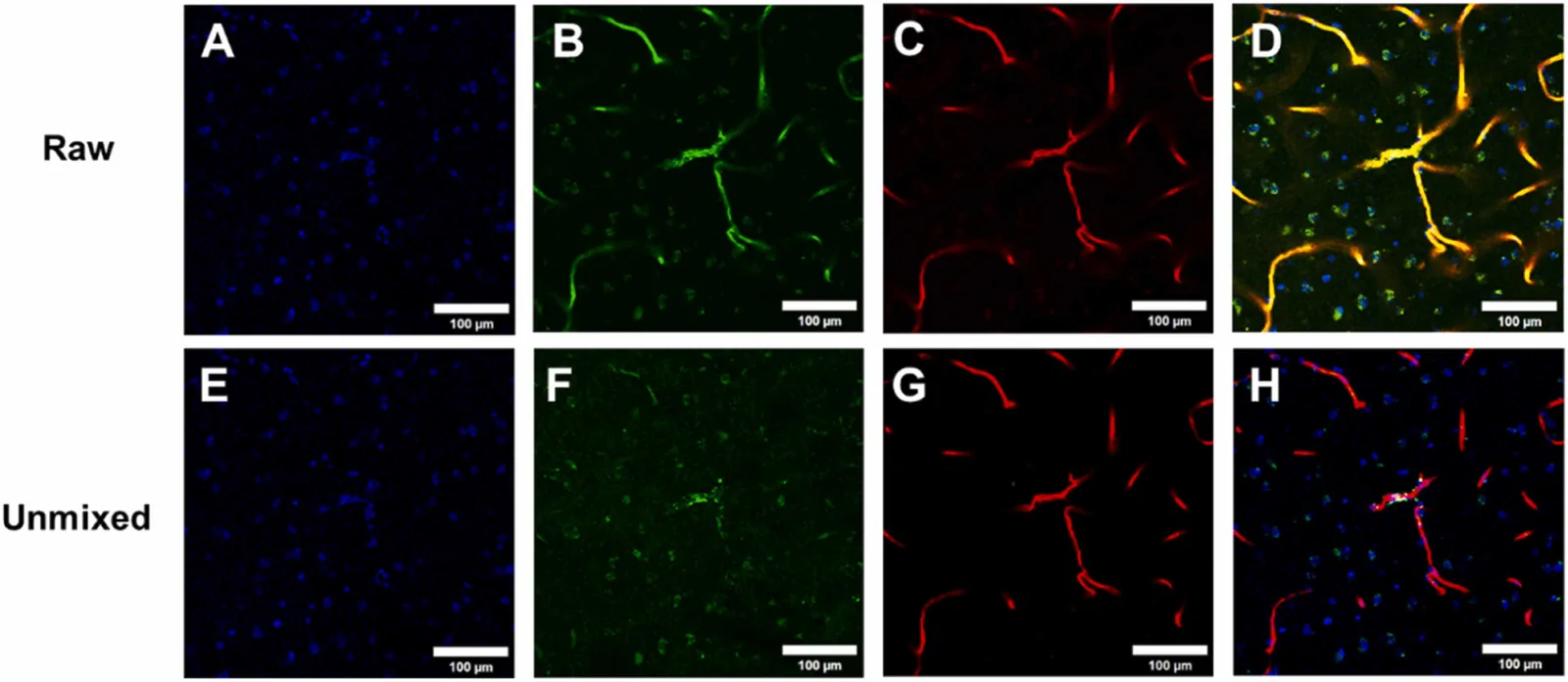
**Spectral unmixing of fluorescence signals in hippocampal tissue from P7.** Top row: raw fluorescence channels prior to unmixing. (A) DAPI channel showing nuclear staining. (B) Green channel capturing lipofuscin autofluorescence, which co-localizes with vascular structures and generates signal overlap. (C) Red channel showing UEA I (DyLight 594) vascular labeling with visible spectral crosstalk from lipofuscin. (D) Composite overlay of raw channels A–C, in which the spectral overlap between lipofuscin and the vascular marker produces a yellow/orange signal along vessel walls. Bottom row: the same field of view after Poisson non-negative matrix factorization (NMF) spectral unmixing. (E) Unmixed DAPI signal. (F) Unmixed lipofuscin autofluorescence, now confined to discrete perinuclear granules. (G) Unmixed vascular signal, showing clearly delineated vessel profiles free of lipofuscin contamination. (H) Composite overlay of unmixed channels E–G, demonstrating effective spectral separation of nuclei (blue), lipofuscin (green), and vasculature (red). Scale bars: 100 μm.

**Fig. 3:**
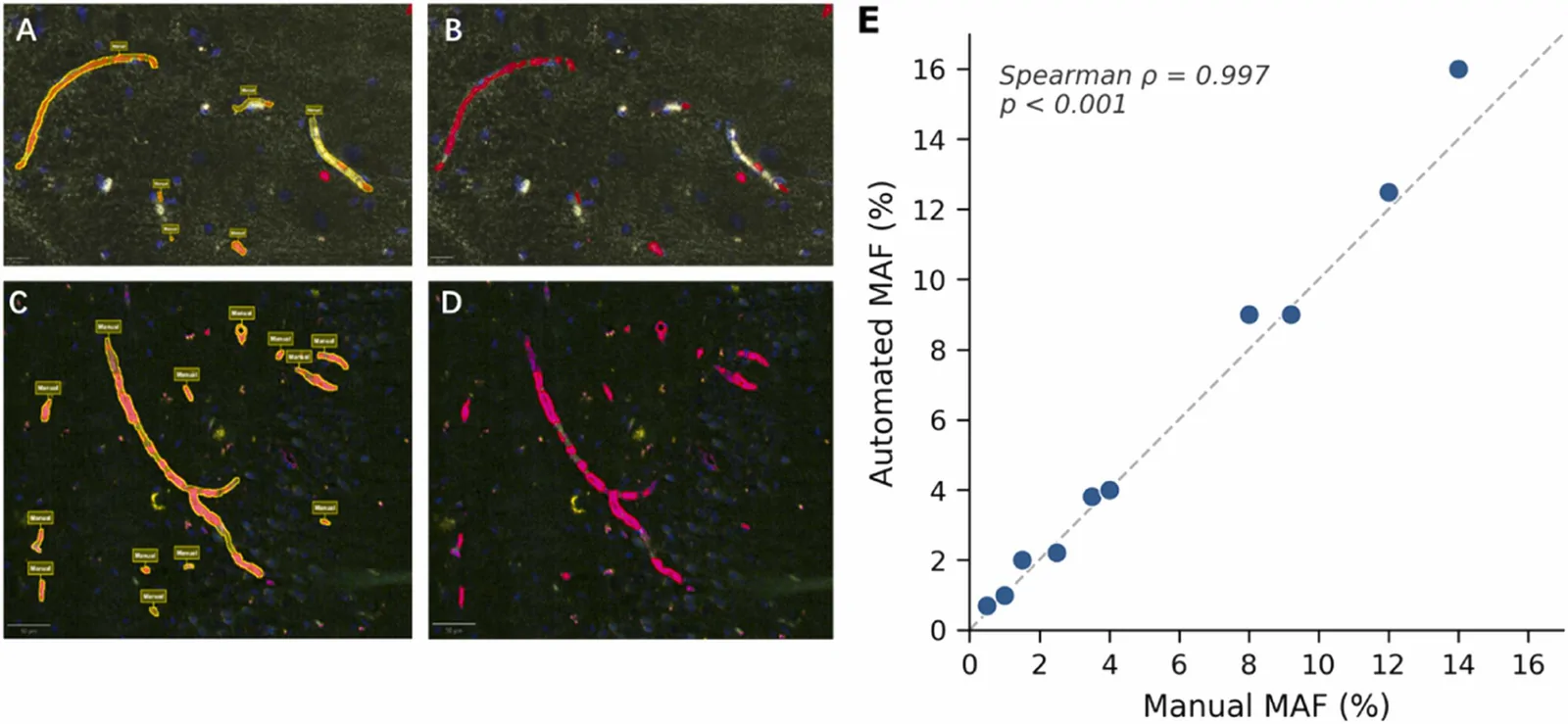
**Validation of the automated quantification workflow.** (A-D) Representative images showing comparison between manual annotation and automated segmentation. (E) Scatter plot illustrating the correlation of MAF between manual ground-truth annotations and the automated workflow across 10 randomized ROIs. The data points cluster tightly along the identity line (dashed). Spearman's rank correlation analysis confirms a statistically significant positive correlation (ρ = 0.997, p < 0.001).

To ensure the reliability of our automated quantification, we validated the segmentation workflow against manual ground-truth annotations in a subset of 10 randomized ROIs. The application of spectral unmixing successfully isolated specific vascular signals from the extensive lipofuscin autofluorescence observed in adult TLE tissue (Fig. 2). Comparison of manual and automated segmentation revealed that the automated algorithm occasionally fragmented long, continuous vessel segments into multiple smaller objects due to local morphological variations, resulting in a slight overestimation of vessel counts in complex vascular networks (Fig. 3A–D). However, the MAF was robust to this fragmentation effect and demonstrated excellent concordance with manual ground-truth annotations (Spearman's ρ = 0.997, p < 0.001; Fig. 3E). MAF was therefore selected as the primary metric for characterizing microvascular structure in subsequent analyses.

### 3.3 Quantitative microvascular analysis and exploratory clinical correlations

**Fig. 4:**
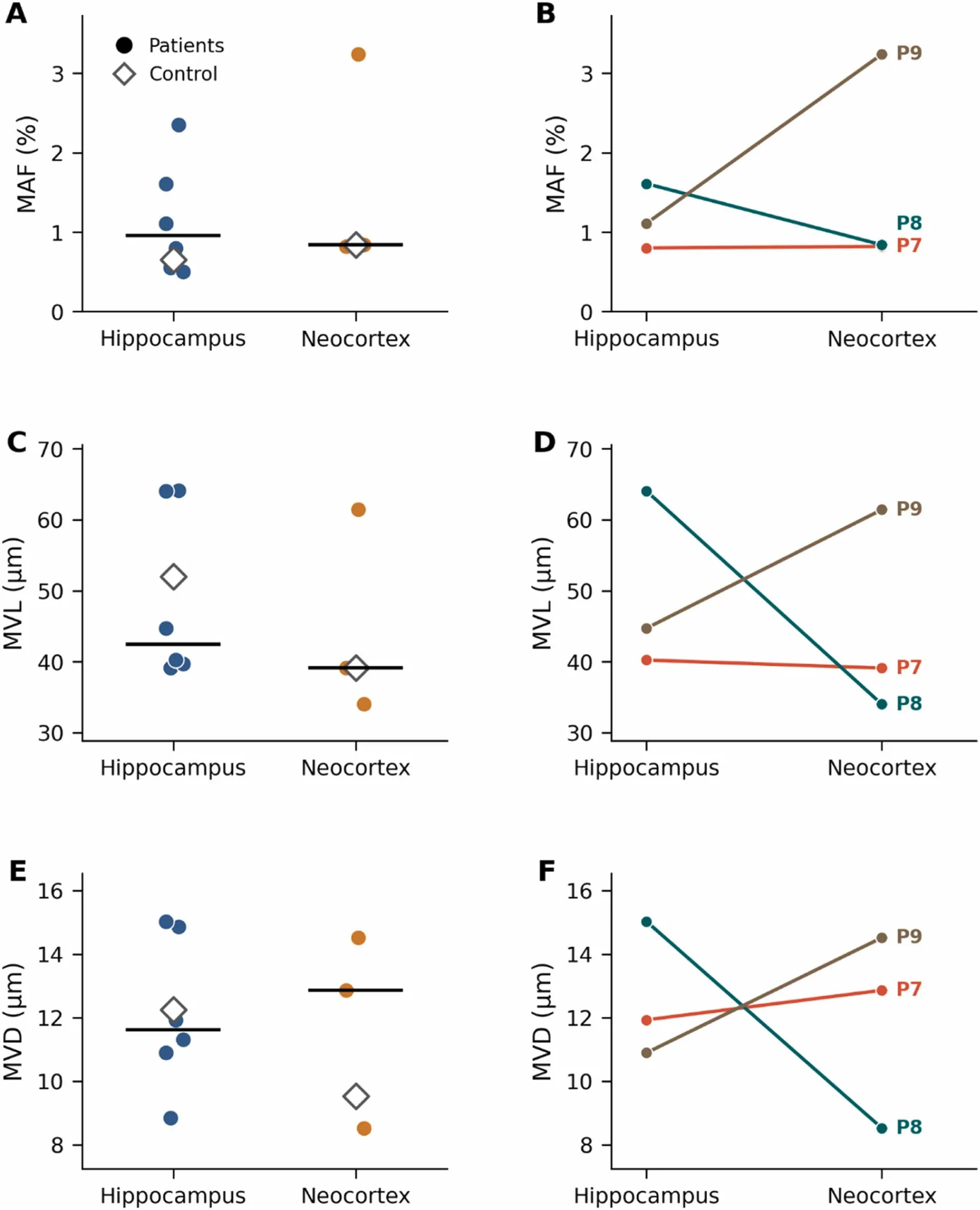
**Structural microvascular metrics in hippocampus and neocortex.** Left column: individual patient values for (A) microvascular area fraction (MAF, %), (C) mean vessel segment length (MVL, μm), and (E) mean vessel diameter (MVD, μm). Each dot represents one patient (hippocampus, n = 6; neocortex, n = 3); the single post-mortem specimen, included as a technical reference, is shown as an open diamond. Horizontal lines indicate medians. Right column: matched-sample slope graphs showing within-patient changes from hippocampus to neocortex for (B) MAF, (D) MVL, and (F) MVD in patients with paired tissue (P7, P8, P9). Each line connects hippocampal and neocortical values from the same patient.

Following validation, the workflow was applied to the available clinical cohort. Quantitative analysis revealed substantial inter-patient heterogeneity in microvascular structural metrics (Fig. 4). For instance, hippocampal MAF ranged from 0.50% to 2.35%, indicating a wide spectrum of vascular density across the TLE cohort. Neocortical samples (n = 3) also showed variability, with MAF values ranging from 0.82% to 3.24%.

**Fig. 5:**
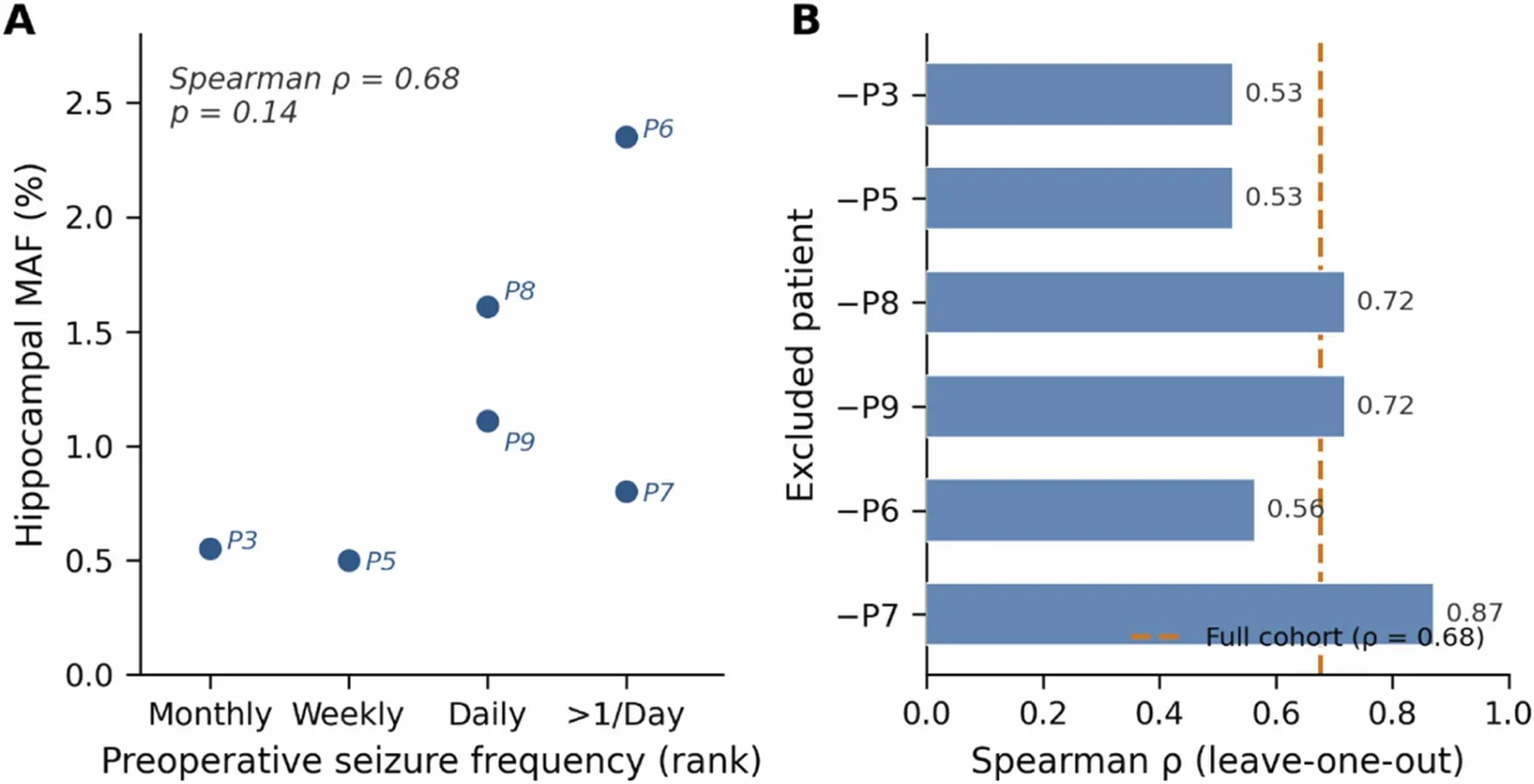
***Exploratory correlation between hippocampal MAF and preoperative seizure frequency.*** Each data point represents one patient (n = 6), labeled by patient identifier. Seizure frequency was ranked on an ordinal scale: Monthly (1/month), Weekly (2–6/week), Daily (1/day), and > 1/Day. Spearman's rank correlation coefficient (ρ = 0.68, p = 0.14) indicated a positive association that did not reach statistical significance. (B) Leave-one-out sensitivity analysis. Each horizontal bar shows the Spearman correlation coefficient recalculated after excluding the indicated patient; the dashed line denotes the full-cohort value (ρ = 0.68). The positive correlation was preserved across all iterations (ρ = 0.53–0.87), including exclusion of the patient with the highest MAF and seizure frequency (P6; ρ = 0.56), indicating that the observed trend is not driven by any single influential observation.

To investigate potential drivers of this hippocampal variability, we performed exploratory Spearman rank correlation analyses between hippocampal MAF and clinical metadata (n = 6). We observed a positive correlation between MAF and preoperative seizure frequency (ρ = 0.68, p = 0.14; Fig. 5), consistent with previous reports linking higher seizure burden to increased microvascular density. A negative correlation was observed between MAF and patient age at surgery (ρ = −0.43, p = 0.40), while a negative correlation was also observed with epilepsy duration (ρ = −0.37). Although none of these correlations reached statistical significance, treating MAF as a continuous variable preserves the granularity of inter-patient differences that would be lost through categorical grouping.

### 3.4 Subfield-resolved microvascular mapping

**Fig. 6:**
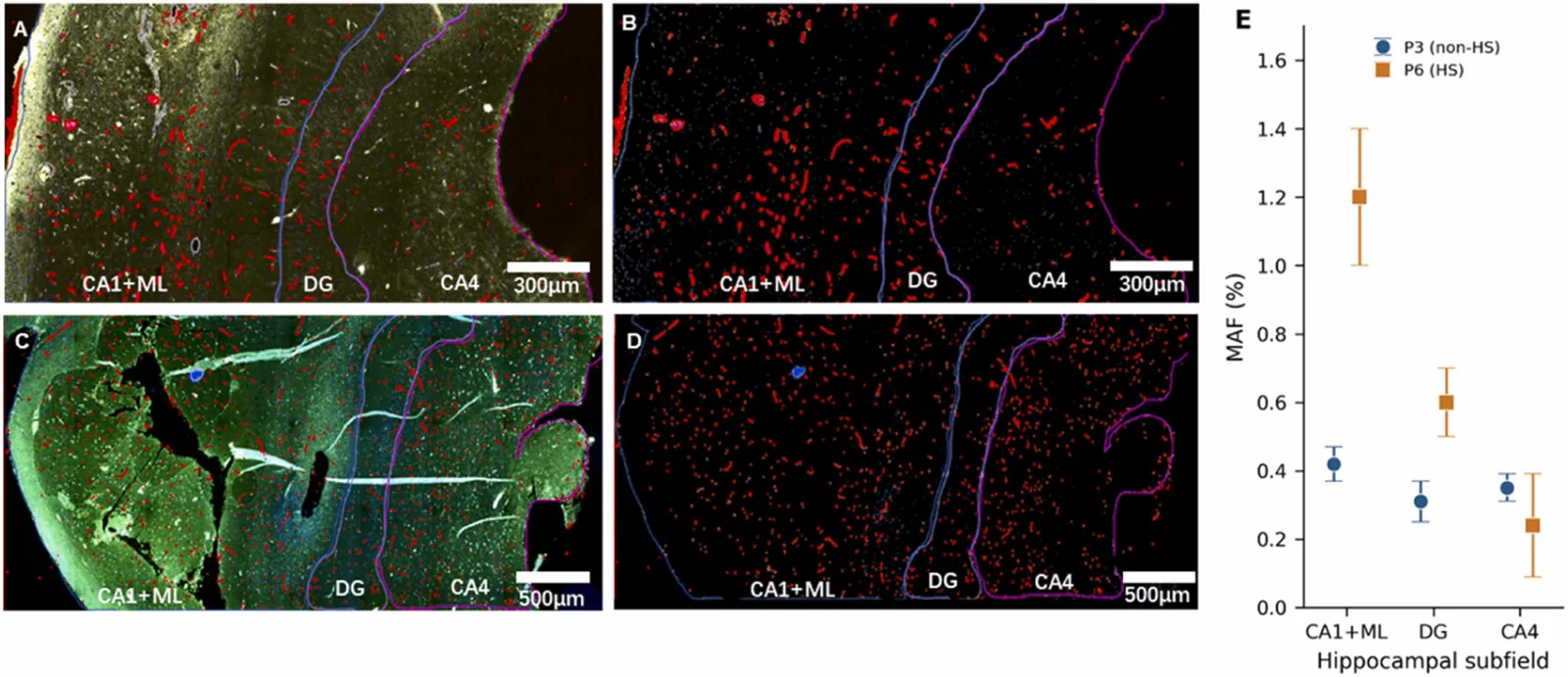
**Subfield-resolved quantification of hippocampal microvasculature.** (A) Fluorescence image with vascular mask overlay (red) from P6 (HS), showing subfield boundaries (white dashed lines) for CA1 +ML, DG, and CA4 delineated using adjacent H&E sections. Scale bar: 300 μm. (B) Isolated vascular mask of the same region as (A), demonstrating the spatial distribution of segmented vessels across subfields. Scale bar: 300 μm. (C) Lower-magnification fluorescence image with vascular mask overlay from P3 (non-HS), with subfield boundaries indicated. Scale bar: 500 μm. (D) Isolated vascular mask of the same region as (C). Scale bar: 500 μm. (E) Subfield-resolved MAF for P6 (HS, orange squares) and P3 (non-HS, blue circles). Error bars represent standard deviation across ROIs within each subfield (n = 3–5 ROIs per subfield). In the HS case, MAF was markedly elevated in CA1 +ML compared to DG and CA4, whereas the non-HS case displayed a relatively uniform distribution across all subfields. Abbreviations: HS, hippocampal sclerosis; MAF, microvascular area fraction; CA1, Cornu Ammonis 1; ML, molecular layer; DG, dentate gyrus; CA4, Cornu Ammonis 4; ROI, region of interest.

Subfield-resolved quantification revealed distinct microvascular patterns across the CA1, CA4, and dentate gyrus regions. In the HS specimen (P6), marked regional variability was detected, with a notably higher MAF in the combined CA1+ML region (1.20%) relative to the DG (0.60%) and CA4 (0.24%) regions (Fig. 6A, B, E). In contrast, the non-HS specimen (P3) displayed a more uniform vascular distribution across all sampled subfields (Fig. 6C, D, E). These case-level observations indicate that heterogeneity in microvascular density is resolvable at the sub-regional level within sclerotic tissue, consistent with known patterns of selective neuronal vulnerability in HS.

## 4 Discussion

Our primary objective was to establish a robust and automated workflow for quantifying microvascular architecture in surgically resected human brain tissue. By integrating H&E-guided anatomical delineation with fluorescence spectral unmixing, we validated a dual-modality pipeline designed to overcome the specific imaging challenges associated with archival human brain specimens. While automated morphometry has been validated in rodent models (Courtney et al., 2021, Todorov et al., 2020), its application to human tissue is frequently confounded by pre-analytical variables such as fixation time and storage duration which can alter antigenicity and tissue autofluorescence (Frigon et al., 2024, Stan et al., 2006). Although our cohort size limited the scope of hypothesis testing, the application of this validated workflow revealed substantial inter-patient heterogeneity in hippocampal MAF. Continuous variable analyses further showed a positive trend between preoperative seizure frequency and hippocampal MAF (van Lanen et al., 2023).

### 4.1 Methodological advancements: beyond mouse models

The drive toward automated whole-slide morphometry has revolutionized quantitative neuropathology. Recent studies have demonstrated the efficacy of open-source platforms such as QuPath for high-throughput quantification of cellular elements in transgenic mouse models (Courtney et al., 2021, Niedowicz et al., 2024). The application of these intensity-thresholding algorithms to adult human tissue is complicated by the accumulation of lipofuscin. This autofluorescent pigment, which accumulates in lysosomes as a result of aging and oxidative stress, emits a broad spectrum that overlaps with common fluorophores (Feldman et al., 2015, Hakvoort et al., 2021). In our dataset, lipofuscin emission interfered with the lectin signal. Standard chemical quenching methods using Sudan Black B or cupric sulfate can reduce this autofluorescence but may also attenuate specific immunofluorescent signals or compromise tissue integrity for downstream analysis (Oliveira et al., 2010, Sapio et al., 2025). Moreover, formalin fixation artifacts lead to increased autofluorescence from red blood cells in the lipofuscin channel, making intensity-based segmentation challenging. To resolve this, we employed spectral unmixing to separate the vascular signal from lipofuscin based on their distinct spectral signatures (Sadashivaiah et al., 2023). This computational clearing approach reduced autofluorescence-related contamination and improved the separability of vascular signals, ensuring that automated segmentation remained robust even in aged specimens with high metabolic waste accumulation (Jiang et al., 2023).

### 4.2 Seizure burden and microvascular remodeling

Our continuous variable analysis suggested a positive trend between preoperative seizure frequency and hippocampal MAF. This observation aligns with previous histopathological studies in human TLE and focal cortical dysplasia which reported that enhanced vascular density correlates with higher seizure frequency, longer epilepsy duration, and earlier age of onset (Greene et al., 2022, Ogaki et al., 2020, Reiss et al., 2023, van Lanen et al., 2021). From a pathophysiological perspective, recurrent ictal activity imposes a significant metabolic demand on the local neural network (Ferlini et al., 2021). The resulting discrepancy between energy supply and demand can create a hypoxic microenvironment which stabilizes Hypoxia-Inducible Factor 1-alpha (HIF-1α) and upregulates downstream angiogenic factors including Vascular Endothelial Growth Factor (VEGF) and erythropoietin (Reiss et al., 2023, Suleymanova and Karan, 2025, van Lanen et al., 2021). The consequential angiogenesis likely represents a compensatory mechanism to restore neurovascular coupling and metabolic support to the epileptogenic zone (Ogaki et al., 2020).

It is important to note that this seizure-induced vascular remodeling may be maladaptive. The newly formed microvessels in epileptic tissue are often morphologically immature and lack a fully functional blood-brain barrier (Reiss et al., 2023). Disrupted barrier integrity permits the extravasation of serum proteins such as albumin into the parenchyma which can trigger TGF-β signaling in astrocytes, impair potassium buffering, and further promote neuronal hyperexcitability and inflammation (Weissberg et al., 2015). Therefore, the elevated MAF observed in patients with high seizure burdens in our cohort likely reflects a pathological cycle of angiogenesis and barrier dysfunction rather than a simple restorative process, a concept supported by the "vascular hypothesis" of drug resistance (Huang et al., 2025, Williams et al., 2019).

To assess the robustness of the observed association between hippocampal MAF and preoperative seizure frequency, we performed a leave-one-out sensitivity analysis. The positive correlation was preserved across all six iterations (Spearman's ρ ranging from 0.53 to 0.87), including the iteration excluding the patient with the highest MAF and seizure frequency (P6; ρ = 0.56). While the association remained non-significant in all iterations, consistent with the limited statistical power of the cohort, the directional stability of the correlation indicates that the observed trend is not driven by any single influential observation. Nevertheless, these exploratory findings require confirmation in adequately powered, pathology-stratified cohorts.

### 4.3 Physiological confounders and subfield-level heterogeneity

Beyond seizure-related pathology, our exploratory analysis highlighted the influence of physiological variables on microvascular structure. We observed a negative correlation coefficient between patient age at surgery and hippocampal MAF. While statistical significance was not reached, this finding aligns with established evidence of age-related microvascular rarefaction characterized by the progressive decline in capillary density, tortuosity, and angiogenic potential in the aging human brain (Brown and Thore, 2011, Mun and Park, 2025, Reeson et al., 2022). Consequently, the high vascular burden observed in younger patients such as the 33-year-old P6 may reflect a cumulative effect of active seizure-induced angiogenesis superimposed upon a preserved youthful vascular reserve. This underscores the necessity for future quantitative studies to stratify cohorts by age to disentangle disease-specific remodeling from physiological aging.

Unlike molecular assays of tissue homogenates (Nicoletti et al., 2008) or whole-region average morphometry (Rigau et al., 2007), our dual-modality approach allows for the quantification of local heterogeneities within distinct anatomical subfields defined by H&E histology (Blümcke et al., 2013). By co-registering fluorescence data with H&E histology, we detected distinct regional heterogeneity that would be obscured in bulk tissue analyses. For instance, in the HS specimen, MAF was notably elevated in the combined CA1+ML region compared with CA4. This pattern is consistent with reports of subfield-specific vulnerability in mesial TLE where cell loss and synaptic reorganization such as mossy fiber sprouting are often accompanied by localized vascular remodeling and glial scarring (Freiman et al., 2021, Middlebrooks et al., 2024). The ability to map these local variations demonstrates that our workflow can capture the spatial complexity of HS and provides a robust tool for investigating how vascular supply matches metabolic demand at the sub-regional level.

### 4.4 Limitations and future directions

The interpretation of these findings requires consideration of several limitations inherent to this exploratory study. First, the restricted sample size of the hippocampal cohort limited our statistical power which prevented definitive hypothesis testing and necessitated the use of non-parametric correlation analyses. While the observed trends regarding seizure frequency and age are biologically plausible, they require validation in larger and pathology-stratified cohorts. Second, our control reference consisted of a single post-mortem tissue specimen, whereas the epilepsy samples were obtained from surgical resections. Although we adhered to standardized fixation protocols, potential differences in tissue processing intervals and the post-mortem interval in control tissue could introduce variability in antigen preservation or vascular morphology (Qiu et al., 2019). Third, while confocal microscopy provides superior optical sectioning, our NMF-based spectral unmixing successfully isolated specific vascular signals using standard widefield epifluorescence. This demonstrates the workflow's high accessibility for routine, high-throughput neuropathology laboratories where expensive confocal systems may not be practical.

Furthermore, the microvascular structural metrics reported here were derived from two-dimensional sections. While this standard histological approach allows for precise H&E-guided subfield mapping, it does not fully capture the three-dimensional topology or tortuosity of the vascular network. The complexity of angio-architecture in the epileptic brain involves branching patterns and loop formations that are best appreciated in volumetric datasets. Future investigations would benefit from the application of advanced volume imaging techniques, such as CUBIC clearing combined with multi-photon microscopy, to resolve these complex networks in three dimensions (Deng et al., 2024, Mai and Lu, 2024, Rusch et al., 2022, Steinman et al., 2017). Crucially, the spectral unmixing workflow established in this study is directly adaptable to such volumetric imaging, offering a necessary solution to eliminate lipofuscin artifacts that typically confound deep-tissue visualization in adult human specimens.

Additionally, integrating these structural readouts with molecular markers of blood-brain barrier integrity such as IgG and fibrinogen would help determine whether the observed increase in vessel density corresponds to functional perfusion or pathological leakage. Furthermore, while our method accurately maps vascular density, future studies incorporating quantitative neuronal loss assessment (e.g., NeuN staining) are necessary to determine the direct spatial correlation between angiogenesis and specific HS subtypes (ILAE Types 1–3).

Finally, validation of the automated workflow against manual annotations was performed exclusively on hippocampal tissue. Given potential differences in cytoarchitecture and lipofuscin distribution between hippocampal and neocortical regions, the accuracy of the pipeline in neocortical specimens remains to be formally established and should be addressed in future work.

An additional methodological consideration concerns the segmentation of the control specimen. Owing to the distinct spectral characteristics of lipofuscin in post-mortem versus surgically resected tissue, NMF-based spectral unmixing did not achieve adequate signal separation in the control, necessitating an alternative machine learning-based segmentation approach (ANN-MLP). Although both pipelines were independently validated against manual annotations, the use of two different segmentation strategies means that the absolute MAF value of the control specimen is not strictly comparable to those derived from the NMF-based workflow applied to the surgical cohort. Accordingly, the control specimen in this study should be regarded as a single technical reference demonstrating workflow adaptability across tissue types, rather than as a normative baseline defining the physiological range of microvascular density. Future studies employing a harmonized segmentation pipeline across a larger set of control specimens will be required to establish reference values for comparative analyses.

## 5 Conclusions

We have established a dual-modality workflow that effectively overcomes lipofuscin artifacts and enables quantification of microvascular architecture in archival human brain tissue. By combining spectral unmixing with H&E-guided anatomical delineation, we revealed inter-patient heterogeneity that is often masked in molecular assays of tissue homogenates or whole-region average morphometry. Our exploratory results suggest possible associations between vascular variability and clinical factors such as preoperative seizure frequency and patient age. These findings provide a methodological foundation for future large-scale, pathology-stratified studies to investigate the role of neurovascular remodeling in chronic epilepsy and its potential as a therapeutic target.

## CRediT authorship contribution statement

**Marc A.M.J. van Zandvoort:** Writing – review & editing, Visualization, Supervision, Project administration, Investigation, Funding acquisition, Conceptualization. **Kim Rijkers:** Writing – review & editing, Visualization, Supervision, Resources, Project administration, Methodology, Data curation. **Olaf E.M.G. Schijns:** Writing – review & editing, Methodology, Data curation. **Rick H.G.J. van Lanen:** Writing – review & editing, Visualization, Resources, Methodology, Formal analysis. **Govert Hoogland:** Writing – review & editing, Visualization, Supervision, Investigation. **Roel H.L. Haeren:** Writing – review & editing. **Steffanie Hartjes:** Methodology, Formal analysis, Data curation. **Alexander Kotenko:** Methodology, Data curation. **Vasiliki Tempeli:** Software, Formal analysis. **Dimitrios Kapsokalyvas:** Writing – review & editing, Visualization, Supervision, Software, Methodology, Formal analysis, Data curation. **Ziyu Xu:** Writing – original draft, Validation, Software, Resources, Methodology, Investigation, Formal analysis, Data curation.

## Consent for publications

All participants (or their legal guardians) provided consent for publication of anonymized research data. No identifiable images or personal data are included in this manuscript.

## Ethical approval and consent to participate

All procedures performed in studies involving human participants were in accordance with the ethical standards of the institutional and/or national research committee and with the 1964 Helsinki declaration and its later amendments or comparable ethical standards. METC approval number: METC azM/UM, ID METC 2018–0664.

## Funding

No funding was received for this research.

## Declaration of Competing Interest

The authors declare that they have no known competing financial interests or personal relationships that could have appeared to influence the work reported in this paper.

## Data Availability

De-identified clinical summaries and derived metrics (MAF/MVL/MVD) are available from the corresponding author upon reasonable request. Restrictions apply to the sharing of raw histology images that may contain potentially identifying metadata.
